# Ethnic minority GP trainees at risk for underperformance assessments: a quantitative cohort study

**DOI:** 10.3399/BJGPO.2022.0082

**Published:** 2022-12-14

**Authors:** Nathanja Mariëtte van Moppes, Sander Willems, Mana Nasori, Jettie Bont, Reinier Akkermans, Nynke van Dijk, Maria van den Muijsenbergh, Mechteld Visser

**Affiliations:** 1 Department of General Practice, Amsterdam UMC, location AMC, Amsterdam, The Netherlands; 2 Quality of Care and Personalised Medicine, Amsterdam Public Health, Amsterdam, The Netherlands; 3 Emergency Department, Rijnstate Hospital, Arnhem, Nijmegen, The Netherlands; 4 Department of Primary and Community Care, Radboud Institute for Health Sciences, Scientific Institute for Quality of Healthcare, Radboud University Medical Center, Radboud University Nijmegen, Nijmegen, The Netherlands; 5 Faculties of Exercise & Sports, Nutrition, and Health, Amsterdam University of Applied Sciences, Amsterdam, The Netherlands; 6 Department of General Practice, Radboud University Medical Center, Radboud University Nijmegen, Nijmegen, The Netherlands

**Keywords:** DEI (diversity, equity, and inclusion), ethnic minority GP trainees, ethnic and racial minorities, GP specialty training, students, general practice

## Abstract

**Background:**

Recent studies suggest that ethnic minority students underperform in standardised assessments commonly used to evaluate their progress. This disparity seems to also hold for postgraduate medical students and GP trainees, and may affect the quality of primary health care, which requires an optimally diverse workforce.

**Aims:**

To address the following: 1) to determine to what extent ethnic minority GP trainees are more at risk of being assessed as underperforming than their majority peers; 2) to investigate whether established underperformance appears in specific competence areas; and 3) to explore first- and second-generation ethnic minority trainees’ deviations.

**Design & setting:**

Quantitative retrospective cohort design in Dutch GP specialty training (start years: 2015–2017).

**Method:**

In 2020–2021, the authors evaluated files on assessed underperformance of 1700 GP trainees at seven Dutch GP specialty training institutes after excluding five opt-outs and 165 incomplete datasets (17.4% ethnic minority trainees). Underperformance was defined as the occurrence of the following, which was prompted by the training institute: 1) preliminary dropout; 2) extension of the educational pathway; and/or 3) mandatory coaching pathways. Statistics Netherlands (CBS) anonymised the files and added data about ethnic group. Thereafter, the authors performed logistic regression for potential underperformance analysis and χ^2^ tests for competence area analysis.

**Results:**

Ethnic minority GP trainees were more likely to face underperformance assessments than the majority group (odds ratio [OR] 2.41, 95% confidence interval [CI] = 1.67 to 3.49). Underperformance was not significantly nested in particular competence areas. First-generation ethnic minority trainees seemed more at risk than their second-generation peers.

**Conclusion:**

Ethnic minority GP trainees seem more at risk of facing educational barriers than the majority group. Additional qualitative research on underlying factors is essential.

## How this fits in

Equitable opportunities for success for ethnic minority GP trainees are essential from a social justice point of view. In addition, research indicates that an ethnically diverse medical workforce is essential for developing cultural competencies required for qualified and accessible health care in an increasingly diverse population. However, recent studies suggest that ethnic minority (postgraduate) students and GP trainees seem at risk of being assessed as underperforming in standardised tests. Scientifically quantifying these suggested differences in assessments encountered by GP trainees from ethnic minority groups is an essential step to acknowledging potential disparities. It encourages additional research on underlying factors and interventions to address these factors. As such, it may foster a personalised and culturally sensitive learning climate that educates GPs to provide qualified and accessible health care to a diverse patient population. This study on GP trainees in the Netherlands demonstrated that belonging to an ethnic minority group was associated with increased odds for assessed underperformance, despite a selective admission procedure ensuring an adequate entrance qualification level for all accepted trainees. Since Dutch GP specialty training has essential similarities in many aspects with international GP training programmes, the results may apply in a broader worldwide context.

## Introduction

The Dutch population is highly diverse. One out of four inhabitants has a migrant background,^1^ and this proportion will grow to 39% in 2060.^2^ United Nations' data^3^ have shown that these figures are not unique to the Netherlands.

GPs will increasingly see patients from different cultures and backgrounds, and research has shown that ethnically diverse student bodies^4^ are essential for developing cultural competencies,^5,6^ and improving healthcare quality and access for underserved population groups.^7,8^

Recent studies suggest that ethnic minority students do not perform well on standardised assessments commonly used to evaluate their academic performance.^9^ A large UK meta-analysis on medical students (*n* = 23 742) showed that candidates from a ‘non-White’ ethnic group often faced underperformance assessments.^10^ Studies on medical students in Australia,^11^ the US,^12,13^ and the Netherlands^14^ demonstrated that the assessed performance of ethnic minority medical students remained behind their majority peers. A comprehensive US review on inclusive educational opportunities indicated ethnically biased assessments and grading disparities.^15^

UK research has shown that these findings may also hold for ethnic minority GP trainees failing specific clinical skills assessments more often than their colleagues from the majority group.^16^ A Dutch interview pilot suggested ethnic minority GP trainees were likely to fail or encounter mandatory coaching pathways.^17^ Yet, quantitative data were lacking to substantiate these findings on eventual discrepancies for ethnic minority GP trainees. This study investigated the extent of potential disparities in assessed performance for Dutch ethnic minority GP trainees, and examined specific competence areas where this underperformance may be nested. Additionally, the study explored possible differences between first- and second-generation ethnic minority GP trainees. In many aspects, the Dutch GP specialty training is comparable with European, British, US, and Australian GP specialty training programmes. Therefore, it is assumed that the results may apply to a broader context.

## Method

### Design

A quantitative retrospective cohort design was used, analysing data from trainee files provided by the GP specialty training institutes.

### Setting

The Dutch GP specialty training is a 3-year dual-track competence-based education aligned with the internationally recognised CanMEDs system (Table 1). GP specialty training in the Netherlands admits 700–800 new trainees annually, allocated to eight training institutes. Approximately 15% of them belong to ethnic minority groups. Second-generation ethnic minority trainees received their pre-training in the Netherlands; first-generation trainees often completed undergraduate degrees abroad. Extensive entry assessments guarantee a high level of knowledge and language. Once admitted, protocolled interventions, such as regular practical observations, systematic test programmes, and reviews of the trainee’s completed learning objectives, support high-quality education.^18,19^ In cases of underperformance, the GP training institutes can prompt the following: 1) removal from the programme; 2) extension of the GP educational pathway; or 3) mandatory coaching pathways.

**Table 1.** Competence areas, corresponding CanMEDs, and description

Competence area	Corresponding CanMEDs	Description
Clinical knowledge and expertise	Medical expert	Interprets the patient's complaints in their context.Applies diagnostic, therapeutic, and preventive ranges that are purposeful and evidence based.
Academic skills	Scholar	Promotes knowledge development and implementation.Facilitates expertise of students, postgraduates, and colleagues.
Communication skills	Communicator	Adequately applies communication techniques and skills.Actively involves patients in the decision-making process.
Organisational skills	Manager	Applies appropriate organisational and management principles.Utilises information technology for optimal patient care.
Teamwork skills	Collaborator	Participates in intra- and interdisciplinary teamwork.Contributes to the health of individual patients and patient groups.
Social accountability	Health advocate	Acts in accordance with legislation, is cost-conscious, and socially involved.
Professional integrity	Professional	Balances personal and professional roles.Works consistently on improving professional skills.

### Participants

Trainees' files were reviewed with starting years 2015, 2016, and 2017. Opt-out emails and advertisements on professional platforms enabled eligible participants to disallow using their data.

### Outcome measures

The outcome measure for the first study question was the relative risk of assessed underperformance. This outcome was operationalised as the occurrence of at least one of the following events: 1) undesired preliminary dropout; 2) extension of the educational pathway; or 3) mandatory coaching pathways.

The second outcome measure was the proportion of assessed underperformance in specific competence areas (Table 1). Notably, assessors could allocate underperformance events to >1 competence area. Also, trainees could face more events of underperformance during their GP training educational pathways.

### Variables

- Minority group: refers to ethnic minorities, following the official definition of CBS, defined as foreign-born (first-generation) or born from at least one foreign-born parent (second-generation).
- Age: expressed in years.
- Sex: male or female.
- GP training institutes: Amsterdam University Medical Center (UMC) (Academic Medical Center [AMC] and VU University Medical Center [VUmc]), UMC Utrecht, Maastricht UMC, Leiden University Medical Center (LUMC), Erasmus Medical Center, Radboud UMC, and UMC Groningen (UMCG).
- Competence areas: see Table 1

### Procedure

Following the European General Data Protection Regulation (GDPR), the Ethical Review Board (ERB) and privacy officers allowed an opt-out procedure before the data sampling. An opt-out link was emailed to all eligible GP trainees and alums with announcements in GP specialty training institutes’ newsletters and at the digital Dutch GP forum (HaWeb). All files of trainees who opted out within 2 months after the aforementioned emails and announcements were excluded.

Thereafter, the data sampling was conducted in three phases.

In phase one, a centralised query collected data from the Dutch GP specialty training institutes’ database on the trainees’ educational start date, age, sex, GP educational institute, and the occurrence of preliminary dropout and extended educational pathways. At this stage, data were not yet completely anonymised since CBS required the associated professional registration numbers to add details on ethnic group.

In phase two, all files were reviewed from trainees who encountered preliminary dropout and/or extended educational pathways to obtain information unavailable in the centralised database on mandatory coaching pathways and specific underperformance areas. These reviews at the regional educational institutes allowed the discussion of doubtful file documentation with local assessors. Subjects of these discussions were as follows: 1) the validity of analysing separate competence areas commonly confused by assessors; and 2) some dropouts apparently by choice, on closer reflection aligned with the institutes' urgent recommendation. Based on these discussions, it was decided that: 1) clustering commonly merged areas would add more value; and 2) dropouts, seemingly on the ambition for a different career, should be considered undesired if they were a result of well-documented insufficient academic progress. Owing to COVID-19 restrictions, not all GP specialty training institutes allowed the researchers to visit their offices physically. Local educational coordinators at the institutes that could not allow visitors supported the research team with the data extraction. To ensure consistent data quality, the research team developed predefined data formats and provided daily online availability for consultations*.*

In phase three, all secured datasets were transferred to CBS, which added details on ethnic group through the birth country of the trainees and their parents and further anonymised the datasets.

### Analysis

The following incomplete cases were excluded:

- cases with incomplete files owing to a remaining training period of ≥3 months (unforeseen underperformance was not expected in the final 3 months of education; therefore, those cases were considered complete for inclusion);
- cases from one GP specialty training institute that could not support the researchers in completing the data through local file reviews; and
- cases with missing key information for CBS to indicate ethnic group.

Selective dropout was checked through an independent *t*-test (for continuous variables) and χ^2^ test (for categorical variables) comparing the population characteristics of the eliminated missing data cases to the complete cases. Descriptive analyses were performed and the following were determined: mean and standard deviation (SD); median and interquartile range for continuous characteristics; and number and percentages for categorical characteristics.

A multilevel logistic regression analysis was performed using a model with a random intercept and fixed variables adjusted for age and sex to examine a potential nesting effect through the hierarchical study structure with trainees nested in training institutes. If this multilevel logistic regression model would not converge and indicate a negligible nesting effect, a single-level logistic regression model would be continued. To assess (clustered) competence areas as a potential field of underperformance, χ^2^ was used. Additionally, possibly different performance outcomes in first- and second-generation migrant trainees were explored using the procedures mentioned above for logistic regression analyses.

All analyses were performed in the highly secured environment of CBS using the Statistical Package for Social Sciences (IBM SPSS Statistics; version 26). A value of *P*<0.05 was considered statistically significant for all analyses based on two-sided testing.

### Ethics

The use of sensitive personal data on ethnic group was inevitable in this study. Measures were taken to protect the integrity of anonymising, transfer, storage, and responsibilities in every possible lawful and ethical way. CBS took responsibility for anonymising and non-traceability to individuals in a secured environment. Only the research team had access to this environment for analysis, and the team could not export data. After completing this study, CBS will keep the data for 10 years in their secured environment to enable scientific verification.

The ERB of the Dutch Association for Medical Education and the privacy officers of all participating institutes carefully reviewed and approved the research protocol based on the GDPR and Dutch legislation. These statutes support opt-out procedures for extensive research populations conditional on a strict focus on solving critical societal issues or re-establishing equal opportunities for potentially underserved populations.

## Results

### Study population

Data were collected on the assessed performance of 1870 trainees from seven Dutch GP specialty training institutes and 170 cases were excluded; five owing to opt-out, 18 because of missing information, and 147 with incomplete educational pathways owing to a remaining training period of ≥3 months. The five opt-outs were eliminated before the analysis and the 165 excluded cases were considered missing cases. A selective dropout analysis showed that GP specialty training institute, ethnic group, and age did not significantly differ from the included cases. A large proportion of the trainees excluded, owing to a remaining training period of ≥3 months, had started their education in 2017 (86%) and were female (91%). Personal circumstances such as maternity leave, illness, or participation in research projects had caused their delays. The small percentage of missing cases (9%), the absence of selective ethnic group dropout, and adjustment for sex and age in the analysis legitimatised a complete-case analysis (CCA) on 1700 included cases and deleting missing cases list-wise.

The ethnic minority trainee percentage was 17.4%. Ethnic minority trainees were more often male (34.1% versus 28.3%, *P*<0.05) and of higher age than the majority group (29.9 years, SD = 4.2 versus 28.6 years, SD = 3.2, *P*<0.001; Table 2).

**Table 2.** Characteristics of the study population

Characteristic	Trainees from the majority group(*n* = 1404, 82.6%)		Trainees from second-generation ethnic minority groups(*n* = 196, 66.2%)		Trainees from first-generation ethnic minority groups(*n* = 100, 33.8%)		Total ethnic minority population(*n* = 296, 17.4%)		*P* value	Total study population (*N* = 1700)	
	*n*	%	*n*	%	*n*	%	*n*	%		*n*	%
**Sex**											
Female	1007	71.7	138	70.4	57	57.0	195	65.9	—	1202	70.7
Male	397	28.3	58	29.6	43	43.0	101	34.1	—	498	29.3
**Age at start GP specialty training**											
Age, years, mean (SD)	—		28.9 (3.6)		31.6 (4.9)		29.9 (4.2)		<0.001	28.9 (3.4)	
**GP specialty training institute**											
1	210	15.0	—	—	—	—	54	18.2	—	264	15.5
2	206	14.7	—	—	—	—	21	7.1	—	227	13.4
3	161	11.5	—	—	—	—	35	11.8	—	196	11.5
4	209	14.9	—	—	—	—	57	19.3	—	266	15.6
5	243	17.3	—	—	—	—	37	12.5	—	280	16.5
6	164	11.7	—	—	—	—	41	13.9	—	205	12.1
7	211	15.0	—	—	—	—	51	17.2	—	262	15.4
**Start year of training**											
2015	501	35.7	66	33.7	37	37.0	103	34.8	—	604	35.5
2016	492	35.0	75	38.3	34	34.0	109	36.8	—	601	35.4
2017	411	29.3	55	28.1	29	29.0	84	28.4	—	495	29.1
**Occurrence of predefined underperformance events**											
Underperformance(≥1 events)	101	7.2	—	—	—	—	53	17.9	<0.001	154	9.1
Mandatory coaching pathway	87	6.2	—	—	—	—	46	15.5	<0.001	133	7.8
Extension of education	69	4.9	—	—	—	—	33	11.1	<0.001	102	6.0
Preliminary dropout^a^	—	—	—	—	—	—	—	—	—	23	1.4

^a^The number of preliminary dropouts prompted by the educational institute in the overall study population was too small to analyse its proportions for ethnic minority and majority trainees without infringing the strict privacy protection rules that applied to this study.

Underperformance events occurred in 154 GP trainees (9.1%) and ethnic minority trainees were significantly overrepresented (17.9% versus 7.2%, *P*<0.001; Table 2). These events included 1.4% of the overall population who preliminary dropped out, 11.1% extended educational pathways among ethnic minority trainees versus 4.9% among majority trainees (*P*<0.001), and 15.5% mandatory coaching pathways among ethnic minority trainees versus 6.2% among majority trainees (*P*<0.001), upon binding advice of the GP training institute. Most trainees (ethnic minority and majority trainees), with underperformance events, experienced ≥1 event.

Additionally, Table 2 shows that second-generation ethnic minority trainees outnumbered first-generation (66.2% versus 33.8%). Male trainees were stronger represented in the first-generation than in the second-generation (43.0% versus 29.6%). The mean age of first-generation ethnic minority trainees was significantly higher than the second-generation (31.6 years, SD = 4.9 versus 28.9 years, SD = 3.6, *P*<0.001).

### Risk of underperformance: ethnic minority versus majority trainees

Differences between the participating GP specialty training institutes could explain only a minor proportion (0.8%) of the outcome variance (intraclass correlation coefficient [ICC] = 0.008). With this non-convergent multilevel regression analysis, it was decided to continue using a single-level analysis.

Table 3 shows that ethnic minority GP trainees were more likely to face underperformance assessments than those from the majority group (OR 2.82, 95% CI = 1.97 to 4.05). When adjusted for age and sex, the OR for underperformance in ethnic minority trainees compared with the majority group was 2.41 (95% CI = 1.67 to 3.49). Higher age (OR 1.10, 95% CI = 1.06 to 1.15) and male sex (OR 1.61, 95% CI = 1.13 to 2.28) were independent risk factors for underperformance.

**Table 3.** Risk of being assessed as underperforming in GP trainees from ethnic minority groups compared with trainees from the majority group (*N* = 1700, logistic regression model without random effects, adjusted for age and sex)

Category	Underperformance odds ratio (95% CI)	*P* value	Standard error
Ethnic minorities	2.82 (1.97 to 4.05)	<0.001	0.18
**Independent risk groups**			
Age	1.10 (1.06 to 1.15)	<0.001	0.02
Sex, male	1.61 (1.13 to 2.28)	0.008	0.18
**Adjusted for age and male sex**			
Ethnic minorities	2.41 (1.67 to 3.49)	<0.001	0.19

### Competence areas

Perspectives of educational coordinators indicated that assessors tended to merge some competence areas. In line with these assessments, the following were clustered: 'clinical knowledge and expertise' with 'academic skills,' and 'organisational skills with teamwork skills' and 'social accountability*'* (Tables 1 and 4). Underperformance assessments in trainees from ethnic minorities were not significantly more often nested in particular (clustered) competence areas (Table 4).

**Table 4.** Insufficiently assessed competence areas in trainees who faced one or more events of underperformance in trainees from ethnic minority groups and the majority group

	Trainees from the majority group(*n* = 1404, 82.6%)		Trainees from ethnic minority groups (*n* = 296, 17.4%)		*P* value	Total study population (*N* = 1700)	
**Category**	***n***	**%**	***n***	**%**		***n***	**%**
Trainees who faced ≥1 events of assessed underperformance	101	7.2	53	17.9	<0.001	154	9.1
**Insufficiently assessed competence areas in trainees who faced underperformance events**							
Communication skills	54	3.8	34	11.5	0.20	88	5.2
Combined (social): Organisational skills Teamwork skills Social accountability	43	3.1	27	9.1	0.32	101	5.9
Professional integrity	67	4.8	34	11.5	0.79	70	4.1
Combined (academic and clinic): Clinical knowledge and expertise Academic skills	50	3.6	29	9.8	0.54	79	4.6

### First-generation ethnic minority trainees versus second-generation ethnic minority trainees

After adjusting for age and sex, first-generation ethnic minority GP trainees (OR 4.02, 95% CI = 2.45 to 6.61) had a greater risk to be assessed as underperforming than second-generation (OR 1.65, 95% CI = 1.01 to 2.68), both compared with majority GP trainees (Table 5).

**Table 5.** Risk of being assessed as underperforming in GP trainees from first- and second-generation ethnic minority groups compared with trainees from the majority group (*N* = 1700, logistic regression model without random effects, adjusted for age and sex)

Category	Underperformance odds ratio (95% CI)	*P* value	Standard error
Second-generation minorities	1.72 (1.06 to 2.77)	<0.001	0.24
First-generation minorities	5.53 (3.45 to 8.88)	0.028	0.25
**Independent risk groups**			
Age	1.09 (1.05 to 1.14)	<0.001	0.02
Sex	1.57 (1.10 to 2.23)	0.012	0.18
**Adjusted for age and male sex**			
Second-generation minorities	1.65 (1.01 to 2.68)	0.045	0.25
First-generation minorities	4.02 (2.45 to 6.61)	<0.001	0.25

## Discussion

### Summary

This study analysed quantitative data on underperformance events of 1700 GP trainees at seven Dutch GP specialty training institutes (17.4% ethnic minority trainees). Ethnic minority trainees were more likely to face underperformance assessments. Moreover, first-generation ethnic minority trainees seemed to be more at risk than their second-generation peers. The study found male and older age to be independent risk factors. There were no significant differences per (clustered) competence area(s).

### Strengths and limitations

Underperformance in GP specialty training is a composite variable, indicated by: 1) formally documented doubts about the trainee’s educational progress by teachers and GP-trainers; and 2) recurrently poor test scores (or low scores in >1 area). In the study setting, most of these teachers’ doubts and performance test results (such as the consultation video test and the Competency Assessment List) were stored in paper files with varying accuracy. Analysing these variables would have led to multiple missing data. Therefore, the outcome (underperformance) was measured by the occurrence of well-documented underperformance events. Still, retrospectively assessing events — even if carefully judged and documented — had its limitations and may have been susceptible to observer bias. This risk was addressed through predefined data formats and daily online consultation availability. Also, the following definitions were extensively discussed with educational coordinators at the local GP training institutes: ‘underperformance-related events’; *‘*competence areas’; and ‘upon the binding advice of the training institute’.

In line with the official CBS definition, this study defined ‘belonging to an ethnic minority’ as foreign-born or born from at least one foreign-born parent. Since the Netherlands has no indigenous minorities, this determination may, from the international point of view, be equivalent to 'people with a migration background'*.* Limiting the definition to the trainees’ or their parents’ country of birth may have failed to do justice to the trainees' self-reported sense of belonging to an ethnic minority group.^20^ This issue was carefully reflected on and it was decided that the Dutch study setting required adhering to the official Dutch terminology.

The Netherlands has always been an immigration country. In many aspects, the Dutch GP specialty training is comparable with most European, British, US, and Australian GP specialty training programmes. It shares characteristics and values (for example, a solid academic basis, 3–4 year dual-track programme, competence-based approach, and longitudinal assessments).^21,22^ Therefore, the Dutch GP specialty training is considered a relevant research setting with results beneficial in a broader context for analysing educational opportunities in ethnic minority populations.

### Comparison with existing literature

Although no significantly different underperformance in specific (clustered) competence areas was found, UK research showed that ethnic minority GP trainees have difficulties in detailed Clinical Skills Assessments.^16^ A Dutch pilot interview study suggested that GP trainees from ethnic minority groups may end up in mandatory coaching pathways or fail more often than their peers from the majority group.^17^ In line with these studies, the results of the present study support the conclusion that ethnic disparities may prevail in GP specialty training.

Previous studies showed that language is critical in written exams and clinical GP communication-based tests. Also, mastering the language of instruction as a second language could lead to passive behaviour in discussions, missing out on essential details, or feelings of not fitting in.^23^ In the present study, most ethnic minority trainees were second-generation and native Dutch speakers. GP specialty training institutes require a high-level professional entrance assessment in Dutch. Although the study did not find any significant differences in assessed communication skills, it is not unthinkable that Dutch as a non-native language and cultural differences (particularly in first-generation ethnic minority trainees) may have played a role.

In a systematic review, Isik *et al* distinguished intrinsic and extrinsic motivational factors for academic growth. Intrinsic factors were self-efficacy, confidence, learning-related emotions, personal characteristics and experiences, and ethnic identity and orientation, while learning climate was an essential extrinsic factor.^24^ Ethnic minority students were more sensitive to both types of motivational factors. Therefore, action research is recommended by the authors of the present study, with interventions focusing on intrinsic and extrinsic motivational factors to enhance equal educational opportunities.

Qualitative studies found feelings of being isolated,^25^ absent academic support networks,^26^ and insufficient understanding of cultural differences in the trainer—trainee relationships^27^ as risk factors.^28,29^ Future qualitative studies on equal academic opportunities should also focus on these issues.

### Implications for research and practice

The low dropout rate of GP trainees combined with a higher mandatory coaching pathway and extended trajectory rate suggests that these interventions could bend the trainees’ learning curve towards successful completion. Nevertheless, performance assessments in this study were significantly different for ethnic minority trainees. Despite the selective admission procedure ensuring an expected shared entrance qualification level, belonging to an ethnic minority group was associated with increased odds for events of assessed underperformance.

Since this study is the first quantitative study in the Netherlands on this subject (to the authors’ knowledge), Dutch GP specialty training institutes have not yet implemented any structured policies for equal opportunities for ethnic minority trainees. Therefore, it is recommended that further qualitative research should be undertaken on underlying factors and undesirable educational barriers (for example, potential assessment bias), followed by participatory research involving all stakeholders (trainees, teachers, tutors, and staff) to develop and implement appropriate interventions for an inclusive learning climate with equitable success opportunities.
