# Correction: Ethnic minority GP trainees at risk for underperformance assessments: a quantitative cohort study

**DOI:** 10.3399/BJGPO.2022.9982

**Published:** 2023-02-08

In the article by van Moppes et al, Ethnic minority GP trainees at risk for underperformance assessments: a quantitative cohort study. BJGP Open 2022; DOI: 10.3399/BJGPO.2022.0082, there was an error in Table 5, under the column ‘Underperformance odds ratio (95% CI)’. The results reported for Second-generation and First-generation minorities were reversed: the result for Second-generation minorities should have been ‘1.72 (1.06 to 2.77)’ and the result for First-generation minorities should have been ‘5.53 (3.45 to 8.88)’. The online version has been corrected.

